# Size of the Optic Nerve Head and Its Relationship with the Thickness of the Macular Ganglion Cell Complex and Peripapillary Retinal Nerve Fiber Layer in Patients with Primary Open Angle Glaucoma

**DOI:** 10.1155/2015/186249

**Published:** 2015-08-03

**Authors:** Nobuko Enomoto, Ayako Anraku, Kyoko Ishida, Asuka Takeyama, Fumihiko Yagi, Goji Tomita

**Affiliations:** Department of Ophthalmology, Toho University, Ohashi Medical Center, 2-17-5 Ohashi, Meguro-ku 153-8515, Japan

## Abstract

*Purpose*. To evaluate the relationships among the optic nerve head (ONH) area, macular ganglion cell complex (mGCC) thickness, circumpapillary retinal nerve fiber layer (cpRNFL) thickness, and visual field defects in patients with primary open angle glaucoma (POAG). *Methods*. This retrospective study included 90 eyes of 90 patients with POAG. The ONH area, rim area, mGCC thickness, and cpRNFL thickness were measured using optical coherence tomography. Mean deviation (MD) was measured using standard automated perimetry. The relationships among clinical factors including age, refraction, the ONH area, the rim area, the mGCC thickness, the cpRNFL thickness, and MD were evaluated using correlation coefficients and multiple regression analyses. *Results*. The significant correlation of the ONH area with refraction (*r* = 0.362, *P* < 0.001), the mGCC thickness (*r* = 0.225, *P* = 0.033), and the cpRNFL thickness (*r* = 0.253, *P* = 0.016) was found. Multiple regression analysis showed that the ONH area, rim area, and MD were selected as significant contributing factors to explain the mGCC thickness and cpRNFL thickness. No factor was selected to explain MD. *Conclusions*. The ONH area, in other words, the disc size itself may affect the mGCC thickness and cpRNFL thickness in POAG patients.

## 1. Introduction

Glaucomatous optic neuropathy is characterized by the progressive loss of retinal ganglion cells and their respective axons, which comprise the retinal nerve fiber layer (RNFL) [1]. With regard to the evaluation of glaucoma, optical coherence tomography (OCT) provides reproducible quantitative measurements of RNFL around the optic nerve head (ONH) [2] and in the macular region [3–7]. RNFL assessment is important because RNFL often precedes functional changes detected by perimetry [8–17].

Conversely, the ONH size is not constant among individuals, with an interindividual variability of approximately 1 : 7 in a normal Caucasian population [18]. The African-American population has a relatively high incidence of glaucoma [19]. The ONH size is reportedly a possible risk factor for glaucomatous optic nerve damage [20–24]. However, several groups have reported no difference in susceptibility between patients with a large disc and those with a small disc [25–30]. Therefore, the relationship between ONH size and glaucomatous optic nerve damage remains controversial. Furthermore, the influence of the ONH size on circumpapillary RNFL (cpRNFL) thickness is not fully understood. Several studies have found that the cpRNFL thickness measured at a fixed diameter is positively correlated with the optic disc area [31, 32]. The implication was that the number of nerve fibers in cpRNFL depends on the disc area and that it might be possible to decrease the variations in the measured cpRNFL thickness if the scan diameter was adjusted according to the disc diameter, although this possibility was not supported by Huang et al. [33] in normal healthy subjects. To the best of our knowledge, there are no published clinical reports evaluating the relationship between the ONH size and the macular ganglion cell complex (mGCC) thickness in eyes with primary open angle glaucoma (POAG). The aim of the present study was to evaluate the relationships among the ONH area, mGCC thickness, and cpRNFL thickness measured using spectral-domain OCT (SD-OCT) and visual field defects in patients with POAG.

## 2. Materials and Methods

### 2.1. Study Participants

This retrospective study was performed in adherence with the guidelines of the Declaration of Helsinki and was approved by the Institutional Review Board (number 13-29) of Toho University, Ohashi Medical Center, Tokyo, Japan. Because of the retrospective nature of this study, the requirement for written informed consent was waived.

We retrospectively reviewed the medical records of 90 eyes of 90 patients with POAG, including normal tension glaucoma (NTG). The patients underwent SD-OCT for measurement of the ONH area, rim area, mGCC thickness, and cpRNFL thickness and standard automated perimetry (SAP) within a 3-month period at our outpatient clinic between May 2008 and September 2012. SAP was performed using the Humphrey Field Analyzer (Humphrey-Zeiss Systems, Dublin, CA, USA) with the 30-2 Swedish Interactive Threshold Algorithm (SITA). The visual field tests were considered reliable when the fixation losses were <20% and the false-positive and false-negative rates were <25%. The mean deviation (MD) was used to assess the severity of visual field loss.

All subjects underwent complete ophthalmological examination and assessment of medical and family histories. The ophthalmological procedures included visual acuity testing with refraction, slit-lamp biomicroscopy, gonioscopy, Goldmann applanation tonometry for the measurement of intraocular pressure (IOP), and dilated stereoscopic fundus examination. The diagnostic criteria for POAG used in this study included the following: normal open anterior chamber angles on slit-lamp biomicroscopy and gonioscopy, presence of a glaucomatous ONH on stereoscopy with corresponding visual field defects, a best-corrected visual acuity of at least 20/25, refractive errors in the spherical equivalent (SE) not exceeding −6 or +3 diopters, and a cylindrical correction of less than 3 diopters. A visual field defect was defined as the presence of three or more significant (*P* < 0.05), contiguous nonedge points with at least one point at *P* < 0.01 level in the pattern deviation plot, along with grading outside the normal limits on the Glaucoma Hemifield test (GHT). Exclusion criteria included the following: a history of intraocular surgery, the presence of intraocular diseases such as diabetic retinopathy or age-related macular degeneration that affect image quality or visual field test results, and a history of systemic diseases such as intracranial disease and/or a history of steroid use, both of which affect IOP and visual field test results. When both eyes were eligible for the study, one eye was randomly selected.

### 2.2. SD-OCT

SD-OCT was performed using the RTVue-100 system (software version 4.0, Optovue Inc., Fremont, CA, USA) to measure the cpRNFL thickness and mGCC thickness. SD-OCT uses a scanning laser diode that emits a beam with a wavelength of 840 ± 10 nm and provides images of ocular microstructures. For all participants, measurements were obtained from both regions on the same day.

In this study, the GCC scanning protocol was used for measuring the mGCC thickness. This protocol includes one horizontal scan and 15 vertical scans that cover 7 mm^2^ region. To achieve the best possible coverage within the temporal region, the GCC protocol centered the scan 1 mm temporal to the center of the fovea. During the total scanning period, 15,000 data points were captured within 0.6 s. The GCC scan creates a 6 mm map, which corresponds to approximately 20° on the visual field map.

The ONH protocol used in this study was designed to measure the cpRNFL thickness and ONH parameters. The total time required to acquire a single scan was 0.55 s. Using the SD-OCT-generated fundus image (video baseline protocol), we were able to manually trace the ONH contour. Using the ONH scanning protocol software, the RNFL thickness was automatically measured at a 3.45 mm diameter around the center of the optic disc. A total of 775 A-scans were obtained under this condition. The ONH scan ring did not pass over peripapillary atrophy. The cpRNFL thickness parameter was designed to evaluate the mean thickness in 360° area. In addition, the ONH protocol includes 12 radial scans measuring 3.4 mm in length (452 A-scans each) and six concentric ring scans measuring 2.5–4.0 mm (587–775 A-scans each), all of which are centered around the optic disc contour line automated by the three-dimensional topographic image protocol. The areas between the A-scans were interpolated and various parameters generated to describe the optic disc. The ONH area and rim area were obtained as disc parameters. Measurements were obtained by a well-trained operator. Data with signal strength index (SSI) values of <30 were excluded.

### 2.3. Statistical Analysis

In this study, the following factors were included for analysis: age, refraction, the ONH area, the rim area, the mGCC thickness, the cpRNFL thickness, and MD. Spearman's rank correlation coefficient was used to evaluate the relationship of the ONH area with other factors. Multiple regression analysis was used to evaluate the relationship of the mGCC thickness or cpRNFL thickness with age, refraction, the rim area, MD, and the ONH area, as well as the relationship of MD with the mGCC thickness, the cpRNFL thickness, age, refraction, the rim area, and the ONH area. All statistical analyses were performed using SPSS statistical software (version 20.0, SPSS Inc., Chicago, IL, USA). Data are expressed as means ± standard deviations (SDs), and a *P* value of < 0.05 was considered statistically significant.

## 3. Results

In the present study, we assessed 90 patients with POAG, including 66 patients (73.3%) with NTG.  Table 1 shows the background characteristics of all patients. Most of the patients had an early stage of glaucoma. The averages of MD were −3.71 ± 3.03 dB.

Table 2 shows Spearman's rank correlation coefficients between the ONH area and age, refraction, the rim area, the mGCC thickness, the cpRNFL thickness, and MD. Three factors were significantly correlated with the ONH area: refraction (*r* = 0.362, *P* < 0.001), the mGCC thickness (*r* = 0.225, *P* = 0.033), and the cpRNFL thickness (*r* = 0.253, *P* = 0.016).

Tables 3 and 4 show the results of multiple regression analysis, wherein the mGCC thickness or cpRNFL thickness was used as the dependent variable and age, refraction, the ONH area, the rim area, and MD were used as explanatory variables. Consequently, the ONH area (slope = 4.283 *μ*m/mm^2^, a standard partial regression coefficient (*β*) = 0.241, 95% confidence interval (CI) = 0.907 to 7.659, and *P* = 0.014), the rim area (slope = 10.329 *μ*m/mm^2^, *β* = 0.386, 95% CI = 5.309 to 15.350, and *P* < 0.001), and MD (slope = 0.569 *μ*m/dB, *β* = 0.207, 95% CI = 0.051 to 1.087, and *P* = 0.032) were selected as significant contributing factors to explain the mGCC thickness. Similarly, the ONH area (slope = 4.394 *μ*m/mm^2^, *β* = 0.231, 95% CI = 0.865 to 7.922, and *P* = 0.015), the rim area (slope = 11.079 *μ*m/mm^2^, *β* = 0.386, 95% CI = 5.832 to 16.327, and *P* < 0.001), and MD (slope = 0.658 *μ*m/dB, *β* = 0.223, 95% CI = 0.117 to 1.200, and *P* = 0.019) were selected as significant contributing factors to explain the cpRNFL thickness.

Table 5 shows the results of multiple regression analysis, wherein MD was used as the dependent variable and age, refraction, the ONH area, the rim area, the mGCC thickness, and the cpRNFL thickness were used as explanatory variables. Consequently, no factor was selected to explain MD.

## 4. Discussion

In the present study, the ONH area was significantly correlated with refraction, the mGCC thickness, and the cpRNFL thickness. With regard to significant correlation between the ONH area and refraction, we acknowledge that this finding was a consequence of the lack of correction for refraction magnification, which is characteristic of the RTVue-100 system. A previous study reported that refraction was correlated with the cpRNFL thickness and mGCC thickness [34] and that RNFL thickness and ONH parameters including disc size measured by SD-OCT are subject to influence from the axial length in normal eyes [35].

The present study, which does not measure the axial length, included 90 patients, with 49 eyes exhibiting refractive errors of less than −3 diopters. Therefore, it is expected that eyes with an axial length longer than the standard were predominant. Accordingly, the fact that a significantly positive correlation was observed between the disc size and refraction indicates the possibility of influence from the axial length, as shown in previous studies [34, 35]. In the future, it is necessary to consider influence of refraction and axial length when we will evaluate the parameters measured using SD-OCT.

With regard to the relationship between the ONH area and the mGCC thickness or cpRNFL thickness, several studies evaluated the mGCC thickness and cpRNFL thickness and reported that the ONH area affects the ability to detect glaucoma [36, 37]. Significant correlations between the rim area and the mGCC thickness and between MD and the mGCC thickness have been also reported [38, 39]. However, no study has ever mentioned a correlation between the ONH area and mGCC thickness. According to Table 3, we found a significant correlation between the mGCC thickness and the ONH area, the rim area, and MD using multiple regression analysis. Although the underlying reason remains unclear, our study suggests that the smaller ONH area, the thinner mGCC thickness.

Similarly, as shown in Table 4, we found a significant correlation between the cpRNFL thickness and the ONH area, the rim area, and MD using multiple regression analysis. Several studies have reported the correlation between the ONH area and the cpRNFL thickness [31, 32, 35]. Savini et al. [31] found a positive correlation between the optic disc size and the 360° RNFL thickness, both of which were measured using OCT. Histological studies also reported a positive correlation between the disc size and number of nerve fibers [40, 41]. However, Huang et al. [33] reported no significant association between the RNFL thickness and the optic disc area. The authors stated that previous publications showing such an association may have been biased by the effects of the axial length on fundus image magnification. Several other investigators found that the cpRNFL thickness was correlated with the axial length and refractive error [35, 42–44]. In the present study, even though patients with high myopia were excluded, a significant correlation was still observed between the ONH area and cpRNFL thickness using multiple regression analysis. Considering the findings of former studies and our results, the ONH area may be related to the cpRNFL thickness.

On the other hand, multiple regression analyses using the mGCC thickness and cpRNFL thickness as objective variables showed corresponding coefficients of determination (*R* ^2^) values of 0.297 (Table 3) and 0.333 (Table 4), respectively. This indicates that the strength of the association was not necessarily high. However, the fact that the disc area was shown as a significant variable in multiple regression analyses including all available factors proves that the disc area itself may affect the mGCC thickness and cpRNFL thickness.

This study also examined the effects of the ONH area, rim area, mGCC thickness, and cpRNFL thickness on visual field defects; no factor was identified as a significant contributing factor to explain MD (Table 5). This result supported those of a meta-analysis [45] that concluded that the ONH area was not an independent risk factor for glaucoma. Previous studies reported significant correlation between the mGCC thickness, cpRNFL thickness, and MD [39, 46]. In most former studies, the enrolled subjects suffered from glaucoma with a mean MD value of less than −7.0 dB from SAP. In contrast, our subjects' mean MD value was −3.7 dB. This means that the subjects in our current study had glaucoma with relatively earlier stages than those in former studies, and these factors may not be well correlated with the visual field defects. Moreover, it was reported that GCC thickness was most useful parameter to evaluate structure and function within the central 10° of macula in glaucoma [47]. Therefore we should further investigate by adding the SITA 10-2 program to visual field tests in future studies.

The strength of the present study was that it revealed, for the first time to our knowledge, the relationship between the disc size and the mGCC thickness. The study also has several limitations. First, because of the retrospective study design, there may be a possible bias in the selection of subjects. Second, we did not have data for the normal subjects for comparison. Third, the magnification was not adjusted for the axial length or refraction. Fourth, it remains unclear whether our findings will be beneficial for glaucoma practice in the future.

In conclusion, the results of the present study suggest that the ONH area is significantly positive correlated with the mGCC thickness and cpRNFL thickness. Furthermore, the ONH area is selected as significant contributing factor to explain the mGCC thickness and cpRNFL thicknesses. Therefore, the disc size itself may affect the mGCC thickness and cpRNFL thickness in eyes with POAG. Further studies are required to provide further evidence supporting our findings and confirm the magnitude of the influence of the disc area on the mGCC thickness and cpRNFL thickness.

## Figures and Tables

**Table 1:** Demographics of the study participants (*n* = 90).

Variable	Total (*n* = 90)
Sex (male/female)	39/51

Age (years)	60.75 ± 11.45 (28–82)

Type (POAG/NTG) (eyes)	24/66

Refractive error (D)	−2.71 ± 2.41 (−5.9–2.3)

ONH area (mm^2^)	2.27 ± 0.47 (1.23–3.73)

Rim area (mm^2^)	0.66 ± 0.31 (0.09–1.79)

mGCC thickness (*μ*m)	77.75 ± 8.34 (58.5–98.7)

cpRNFL thickness (*μ*m)	81.79 ± 8.95 (61.15–99.98)

MD (dB)	−3.71 ± 3.03 (−11.31–1.45)

Mean ± standard deviation (range).

POAG: primary open angle glaucoma, NTG: normal tension glaucoma, D: diopter, ONH: optic nerve head, mGCC: macular ganglion cell complex, cpRNFL: circumpapillary retinal nerve fiber layer, and MD: mean deviation.

**Table 2:** Correlations of the ONH area with other factors.

	*r*	*P* value
Age	0.173	0.102
Ref.	0.362	**<0.001**
Rim area	−0.089	0.406
mGCC thickness	0.225	**0.033**
cpRNFL thickness	0.253	**0.016**
MD	0.139	0.192

*r*: Spearman's rank correlation coefficient.

ONH: optic nerve head, Ref.: refractive errors in the spherical equivalent, mGCC: macular ganglion cell complex, cpRNFL: circumpapillary retinal nerve fiber layer, and MD: mean deviation.

**Table 3:** Multiple regression analysis for the relationship between the mGCC thickness and other factors.

	Slope	SE	*β*	95% CI	*P* value
Age	−0.113	0.072	−0.155	−0.256, 0.030	0.120
Ref.	0.314	0.361	0.091	−0.403, 1.031	0.386
ONH area	4.283	1.698	0.241	0.907, 7.659	**0.014**
Rim area	10.329	2.525	0.386	5.309, 15.350	<**0.001**
MD	0.569	0.261	0.207	0.051, 1.087	**0.032**

mGCC: macular ganglion cell complex, SE: standard error, *β*: standardized partial regression coefficient, CI: confidence interval, Ref.: refractive errors in the spherical equivalent, ONH: optic nerve head, and MD: mean deviation.

**Table 4:** Multiple regression analysis for the relationship between the cpRNFL thickness and other factors.

	Slope	SE	*β*	95% CI	*P* value
Age	−0.127	0.075	−0.162	−0.276, 0.023	0.096
Ref.	0.688	0.377	0.185	−0.061, 1.438	0.071
ONH area	4.394	1.774	0.231	0.865, 7.922	**0.015**
Rim area	11.079	2.639	0.386	5.832, 16.327	<**0.001**
MD	0.658	0.272	0.223	0.117, 1.200	**0.019**

cpRNFL: circumpapillary retinal nerve fiber layer, SE: standard error, *β*: standardized partial regression coefficient, CI: confidence interval, Ref.: refractive errors in the spherical equivalent, ONH: optic nerve head, and MD: mean deviation.

**Table 5:** Multiple regression analysis for the relationship between MD and other factors.

	Slope	SE	*β*	95% CI	*P* value
Age	0.001	0.003	0.003	−0.058, 0.060	0.980
Ref.	<0.001	0.148	<0.001	−0.294, 0.295	0.997
ONH area	0.065	0.720	0.010	−1.368, 1.498	0.929
Rim area	1.415	1.150	0.145	−0.872, 3.703	0.222
mGCC thickness	0.063	0.047	0.174	−0.029, 0.156	0.178
cpRNFL thickness	0.075	0.044	0.221	−0.013, 0.163	0.094

MD: mean deviation, SE: standard error, *β*: standardized partial regression coefficient, CI: confidence interval, Ref.: refractive errors in the spherical equivalent, ONH: optic nerve head, mGCC: macular ganglion cell complex, and cpRNFL: circumpapillary retinal nerve fiber layer.
