# Erratum to “Size of the Optic Nerve Head and Its Relationship with the Thickness of the Macular Ganglion Cell Complex and Peripapillary Retinal Nerve Fiber Layer in Patients with Primary Open Angle Glaucoma”

**DOI:** 10.1155/2015/747302

**Published:** 2015-10-07

**Authors:** Nobuko Enomoto, Ayako Anraku, Kyoko Ishida, Asuka Takeyama, Fumihiko Yagi, Goji Tomita

**Affiliations:** Department of Ophthalmology, Toho University, Ohashi Medical Center, 2-17-5 Ohashi, Meguro-ku 153-8515, Japan

In the paper titled “Size of the Optic Nerve Head and Its Relationship with the Thickness of the Macular Ganglion Cell Complex and Peripapillary Retinal Nerve Fiber Layer in Patients with Primary Open Angle Glaucoma,” [1] there was a typographical error in Table  5. The value of SE/age in Table  5 should be written as 0.030. Corrected Table 5 is shown.

This correction does not change any conclusion or findings of the paper.

## Figures and Tables

**Table 1:** Multiple regression analysis for the relationship between MD and other factors.

	Slope	SE	*β*	95% CI	*P* value
Age	0.001	0.030	0.003	−0.058, 0.060	0.980
Ref.	<0.001	0.148	<0.001	−0.294, 0.295	0.997
ONH area	0.065	0.720	0.010	−1.368, 1.498	0.929
Rim area	1.415	1.150	0.145	−0.872, 3.703	0.222
mGCC thickness	0.063	0.047	0.174	−0.029, 0.156	0.178
cpRNFL thickness	0.075	0.044	0.221	−0.013, 0.163	0.094

MD: mean deviation, SE: standard error, *β*: standardized partial regression coefficient, CI: confidence interval, Ref.: refractive errors in the spherical equivalent, ONH: optic nerve head, mGCC: macular ganglion cell complex, and cpRNFL: circumpapillary retinal nerve fiber layer.
